# Arsenic trioxide ameliorates experimental autoimmune encephalomyelitis in C57BL/6 mice by inducing CD4^+^ T cell apoptosis

**DOI:** 10.1186/s12974-020-01829-x

**Published:** 2020-05-06

**Authors:** Ke An, Meng-Jiao Xue, Jia-Ying Zhong, Sheng-Nan Yu, Tian-Shu Lan, Zhong-Quan Qi, Jun-Jie Xia

**Affiliations:** 1 Organ Transplantation Institute of Xiamen University, Fujian Provincial Key Laboratory of Organ and Tissue Regeneration, School of Medicine, Xiamen University, Xiamen, Fujian China; 2 Department of Obstetrics and Gynecology, Zhongshan Hospital, Xiamen University, Xiamen, Fujian China; 3 Key Laboratory of Functional and Clinical Translational Medicine, Fujian Province University, Xiamen Medical College, Xiamen, Fujian China; 4 School of Medicine, Guangxi University, Nanning, Guangxi China

**Keywords:** Experimental autoimmune encephalomyelitis, Arsenic trioxide, CD4^+^ T cells, Apoptosis

## Abstract

**Background:**

Multiple sclerosis (MS) is an immune-mediated disease of the central nervous system characterized by severe white matter demyelination. Because of its complex pathogenesis, there is no definite cure for MS. Experimental autoimmune encephalomyelitis (EAE) is an ideal animal model for the study of MS. Arsenic trioxide (ATO) is an ancient Chinese medicine used for its therapeutic properties with several autoimmune diseases. It is also used to inhibit acute immune rejection due to its anti-inflammatory and immunosuppressive properties. However, it is unclear whether ATO has a therapeutic effect on EAE, and the underlying mechanisms have not yet been clearly elucidated. In this study, we attempted to assess whether ATO could be used to ameliorate EAE in mice.

**Methods:**

ATO (0.5 mg/kg/day) was administered intraperitoneally to EAE mice 10 days post-immunization for 8 days. On day 22 post-immunization, the spinal cord, spleen, and blood were collected to analyze demyelination, inflammation, microglia activation, and the proportion of CD4^+^ T cells. In vitro, for mechanistic studies, CD4^+^ T cells were sorted from the spleen of naïve C57BL/6 mice and treated with ATO and then used for an apoptosis assay, JC-1 staining, imaging under a transmission electron microscope, and western blotting.

**Results:**

ATO delayed the onset of EAE and alleviated the severity of EAE in mice. Treatment with ATO also attenuated demyelination, alleviated inflammation, reduced microglia activation, and decreased the expression levels of IL-2, IFN-γ, IL-1β, IL-6, and TNF-α in EAE mice. Moreover, the number and proportion of CD4^+^ T cells in the spinal cord, spleen, and peripheral blood were reduced in ATO-treated EAE mice. Finally, ATO induced CD4^+^ T cell apoptosis via the mitochondrial pathway both in vitro and in vivo. Additionally, the administration of ATO had no adverse effect on the heart, liver, or kidney function, nor did it induce apoptosis in the spinal cord.

**Conclusions:**

Overall, our findings indicated that ATO plays a protective role in the initiation and progression of EAE and has the potential to be a novel drug in the treatment of MS.

## Background

Multiple sclerosis (MS) is an immune-mediated disease of the central nervous system (CNS) that is typically characterized by neuroinflammation, demyelinated white matter, gliosis, and blood-brain barrier damage [1]. MS onset primarily occurs between the ages of 20 and 40 years, and is the second major neurological disease to cause disability in young people [2, 3]. Although the pathogenic mechanisms underlying MS remains unclear, T cells, B cells, and other immune cells appear to mediate immune responses in the CNS [1]. Currently, at least 19 disease-modifying therapies (DMTs) have been approved for treatment of MS in the USA, all of which can reduce rates of relapse to some extent by exerting immunosuppressive or immunomodulatory effects. However, current drugs are only effective for some MS patients and have no therapeutic action against secondary progressive MS. Additionally, the long-term effects of these treatments are associated with complications [4]. Consequently, there is an urgent need to develop a novel and effective therapeutic drug.

Experimental autoimmune encephalomyelitis (EAE) induced by immunization of C57BL/6 mice with myelin oligodendrocyte glycoprotein (MOG_35–55_) is an ideal model to study MS [5, 6]. It has been reported that Th1 cells influence the initiation of EAE pathogenesis [7]. When the local cytokine environment contains IL-12 and IFN-γ, naïve CD4^+^ T cells can be selectively induced to express T-bet and further differentiate into Th1 cells to activate macrophages contributing to cellular immunity by secreting IFN-γ, IL-2, and TNF-α [8].

Arsenic is a metalloid element that exists widely in nature in different forms and oxidation states. The primary arsenics found in air, soil, sediment, and water are inorganic arsenics, such as arsenite and arsenate. However, organic arsenics, such as arsenobetaine and arsenosugars, are found in seafood. In general, inorganic arsenic is more toxic than organic arsenic [9]. The trivalent arsenic is the most toxic and often interacts with thiol-containing enzymes, suppressing critical biochemical reactions, while the pentavalent arsenic is less toxic but usually replaces the phosphate group in some metabolic pathways [10]. Organic arsenic is mildly toxic, and even some arsenics with a higher degree of alkylation, such as arsenobetaine and arsenosugars, are almost non-toxic. Additionally, arsenic-based products have been used as food additives [11] or pesticides [12] for decades.

Arsenic trioxide (ATO) is an ancient Chinese medicine used to treat several diseases [13]. Studies have reported that ATO can treat syphilis and trypanosomiasis, which both damage the CNS [14, 15]. In the 19th century, ATO was successfully approved as the frontline agent for the treatment of acute promyelocytic leukemia (APL) [16]. In APL patients, ATO promotes the degradation of the promyelocytic leukemia protein/retinoic acid receptor-alpha fusion protein that drives the growth of APL cells, leading to apoptosis and partial differentiation of APL cells [17]. Studies have also shown that ATO exerts therapeutic effects against various solid tumor cells such as breast cancer, ovarian cancer, hepatoma, prostate cancer, pancreatic cancer, and gastric cancer [13]. Although the exact underlying mechanisms are not entirely understood, ATO may induce apoptosis, promote cell differentiation, suppress cell growth, and inhibit angiogenesis in many different tumor cell lines [13].

Recently, it has been shown that ATO is a novel and efficacious therapeutic drug in the treatment of autoimmune diseases, such as asthma [18] and human lupus-like syndrome [19]. A recent study has suggested that ATO suppresses acute graft-versus-host disease in mice [20]. Our previous work has demonstrated that ATO attenuates acute rejection and prolongs graft survival in heart [21] and islet [22] transplantation models. These findings indicate that ATO elicits anti-inflammatory and immunosuppressive effects. However, it is not clear whether ATO has therapeutic effects in EAE.

In this study, we assessed the therapeutic effects of ATO in EAE mice by evaluating differences in clinical symptoms, histology and microglial activation in the spinal cord, expression levels of inflammatory factors, and proportion of CD4^+^ T cells between ATO-treated and non-treated mice. Additionally, we also investigated the underlying mechanism of the ameliorating effects of ATO in EAE mice.

## Methods

### Mice

Female C57BL/6 mice (6–8 weeks old, 20 ± 2 g) were purchased from Vitallihua Experimental Animal Co., Ltd. (Beijing, China). All mice were housed in a specific pathogen-free facility. All experiments in this study were approved and performed in accordance with the guidelines of the Animal Ethics Committee of Xiamen University (approval ID: XDYX2015008).

### ATO

Arsenious acid and sodium chloride for injection was purchased from Harbin Medical University Pharmaceutical Co., Ltd. (Harbin, China) with an ATO concentration of 1 mg/mL. A working solution was prepared for in vitro and in vivo experiments by diluting the injection in normal saline.

### EAE induction and ATO treatment

To induce EAE, female C57BL/6 mice were immunized with 200 μg of MOG_35–55_ peptide (BAM Biotech Co., Ltd., Xiamen, China) in complete Freund’s adjuvant (Sigma, MO, USA) supplemented with 2.5 mg/mL H37RA (Cohesion Biosciences, CA, England). Pertussis toxin (500 ng; List Biological Laboratories Inc., CA, USA) was administered intraperitoneally on the day of immunization and 48 h later. The clinical symptoms were scored as follows: 0, normal; 1, tail paralysis; 2, partial hindlimb paralysis; 3, complete hindlimb paralysis; and 4, complete hindlimb paralysis and partial forelimb weakness. Animals were randomly divided into three groups (10 mice/group): control, no treatment; EAE, MOG treatment; and EAE + ATO (MOG combined ATO treatment). Starting at 10 days post-immunization, mice in the EAE + ATO group were intraperitoneally injected with ATO (0.5 mg/kg/day) for 8 days. On day 22 post-immunization, mice were sacrificed and peripheral blood, spinal cord, and spleen were collected and used for further experiments.

### Histopathology, immunohistochemistry, and immunofluorescence

The spinal cord was dissected and fixed with ice-cold 4% paraformaldehyde overnight at 4 °C, embedded in paraffin, cut into 5 μm slices, and stained with luxol fast blue (LFB) and hematoxylin and eosin (HE). LFB-stained sections were scored for demyelination as follows: 0, none; 1, rare foci; 2, a few areas of demyelination; 3, one to two large areas of demyelination; and 4, extensive demyelination. Representative examples of LFB-stained histological sections illustrating the different demyelination scores are presented in Supplementary Figure 1. HE-stained sections were also scored for inflammation as follows: 0, none; 1, a few scattered inflammatory cells; 2, perivascular infiltrates; 3, extensive perivascular cuffing with extension into adjacent parenchyma; and 4, extensive cell infiltration in white matter [23]. Additionally, the sections were subjected to indirect immunostaining. The primary antibodies were used as follows: anti-CD4 (1:70, Servicebio, Wuhan, China), anti-MBP (1:50, Boster, Wuhan, China), and anti-Iba-1 (1:200, Servicebio). Staining was quantified using HALO™ image analysis software (Indica Labs, NM, USA). Briefly, in the HALO analysis software, we set the measurement target area for each slice. For analysis of CD4^+^ cells in the spinal cord, the Indica Labs-Multiplex IHC module was used to set Stain 1 as negative cells (blue nuclei) and Stain 2 as positive cells (brown granule). For the analysis of Iba-1^+^ cells in the spinal cord, the Indica Labs-HighPlex FL module was used to identify positive cells (red cytoplasm) and negative cells (blue nuclei). After the setup was completed, the software automatically calculated the number of positive and total cells on the section. The percentage of positive cells among all cells was calculated from nine different sections of the spinal cord.

### Proinflammatory cytokine detection

Blood was collected from the cavernous sinus in the posterior eye orbit and kept at room temperature for 30 min. After centrifugation at 4000 rpm for 20 min, the serum was transferred into a new tube and stored at − 80 °C. The concentration of IFN-γ in serum was measured using a commercial V-PLEX proinflammatory panel 1 kit (MSD, NJ, USA).

### Flow cytometry

Red blood cells were removed to obtain peripheral and spleen lymphocytes. The cells were incubated in anti-CD4-FITC (BioLegend, CA, USA) at 4 °C for 30 min. The IgG-FITC isotype antibody (Biolegend) was used as the negative control. The stained cells were examined by flow cytometry with Beckman Cytoflex S (CA, USA). All data resulting from flow cytometry were processed using FlowJo software V.10.

### Assessment of liver and kidney function parameters

ALT (alanine aminotransferase), AST (aspartic aminotransferase), creatinine, and urea in serum were measured using a Mindray automated biochemical analyzer (Mindray Bio-medical Electronics Co., Ltd., Shenzhen, China) using standard diagnostic kits and an analytical grade reagent (Mindray, Shenzhen, China) according to the manufacturer’s instructions.

### Apoptosis

CD4^+^ T cell apoptosis was detected using the Annexin V-FITC/PI Detection Kit (Meilunbio, Dalian, China). Briefly, after washing with PBS, cells were stained with Annexin V-FITC and PI for 15 min at room temperature. Subsequently, cells were detected by flow cytometry with Beckman Cytoflex S. Apoptosis signals of spinal cords were examined with the Apoptosis Detection Kit (Servicebio).

### JC-1 staining

CD4^+^ T cells were sorted from the spleen of naïve C57BL/6 mice using the Mouse CD4^+^ T cell Isolation Kit (Miltenyi, Bergisch Gladbach, Germany), seeded in 12-well plates, and treated with 5 μg/mL ConA alone or 5 μg/mL ConA + ATO (1 and 2 μM) for 24 h. JC-1 staining was performed to monitor the change of mitochondrial membrane potential. Briefly, the CD4^+^ T cells were incubated with 1640 RPMI medium containing 10 mg/mL JC-1 probe (Sigma) for 30 min at 37 °C. After washing three times with PBS, the stained cells were examined by flow cytometry with Beckman Cytoflex S.

### Transmission electron microscope (TEM)

CD4^+^ T cells were cultured and treated as described above. After washing with PBS, the cells were fixed with 2.5% glutaraldehyde overnight at 4 °C. The following day, they were fixed again with 1% osmium tetroxide for 2.5 h at room temperature. The cells were then embedded after being dehydrated. Ultra-thin sections were counterstained with uranyl acetate for 30 min and lead citrate for 30 s and observed under a transmission electron microscope (TEM) (HT7800, Hitachi).

### Caspase 3 activity assays

Total protein was extracted from CD4^+^ T cells to measure Caspase 3 activity with the Caspase 3 Activity Assay kit (Applygen, Beijing, China). Briefly, a BCA assay was used to determine the protein concentrations; next, 10 μL of Caspase 3 substrate was incubated with 10 μL of the total protein (30 μg) in a final volume of 100 μL for 3 h at 37 °C. The absorbance of *p*-nitroanilide was measured using a microplate reader (Thermo Fisher Scientific, Waltham, MA, USA) at 405 nm in turn to calculate the Caspase 3 activity.

### Quantitative real-time PCR (qRT-PCR)

Total RNA was extracted from spinal cords or spleen with Trizol (TansGen, Beijing, China). Reverse transcription was performed using the cDNA Synthesis SuperMix for qPCR kit (TansGen). The mRNA expression levels were quantified using qPCR SuperMix kit (TansGen) and normalized to β-actin. The relative mRNA expression levels of genes were calculated using the 2^−ΔΔCt^ method. ΔΔCt = (*C*_T, target_ – mean *C*_T, β-actin_) _treated sample_−(mean *C*_T, target_ – mean *C*_T, β-actin_) _control sample_. The control sample, from the spinal cord of naïve C57BL/6 mice, was used as the calibrator. The sequences of primers are as follows: IL-1β, forward 5′-TCGCAGCAGCACATCAACAAGAG-3′, reverse 5′-TGCTCATGTCCTCATCCTGGAAGG-3′; IL-2, forward 5′-GGAGCAGCTGTTGATGGACCTAC-3, reverse 5′-AATCCAGAACATGCCGCAGAG-3′; IL-6, forward 5′-TGGGACTGATGCTGGTGACA-3′, reverse 5′-ACAGGTCTGTTGGGAGTGGT-3′; IFN-γ, forward 5′-CGGCACAGTCATTGAAAGCCTA-3′, reverse 5′-GTTGCTGATGGCCTGATTGTC-3′; TNF-α, forward 5′-GCCTCTTCTCATTCCTGCTTGTGG-3′, reverse 5′-GTGGTTTGTGAGTGTGAGGGTCTG-3′; MBP, forward 5′-GCTCTGGCAAGGACTCACACAC-3′, reverse 5′-TGGAGGTGGTGTTCGAGGTGTC-3′; β-actin, forward 5′-CATCCGTAAAGACCTCTATGCCAAC-3′, reverse 5′-ATGGAGCCACCGATCCACA-3′.

### Western blot

Spinal cords were homogenized and CD4^+^ T cells were lysed with cold RIPA buffer (Proteintech, IL, USA) supplemented with a protease inhibitor cocktail (Sigma). After centrifugation at 4 °C for 15 min, the supernatants were collected and used for western blot analysis. The following antibodies were used: anti-Bax (1:1,000, Proteintech, IL, USA), anti-MBP (1:1,000, Proteintech), anti-cleaved Caspase 3 (1:1,000, Affinity, OH, USA), anti-cleaved Caspase 9 (1:1,000, Affinity), anti-Bcl-2 (1:1,000, Proteintech), anti-cleaved PARP (1:1,000, Affinity), and anti-β-actin (1:5,000, Bioworld, MN, USA).

### Statistical analysis

Data were analyzed with GraphPad Prism 6 software (La Jolla, CA, USA) and represented as means ± SD of three separate experiments. Clinical scores, demyelination scores, and inflammation scores were compared using the Kruskal–Wallis test. A one-way ANOVA was used for multiple comparisons in the rest of the assays. A *p* value<0.05 was considered to be statistically significant.

## Results

### ATO ameliorated EAE progression in mice

**Fig. 1:**
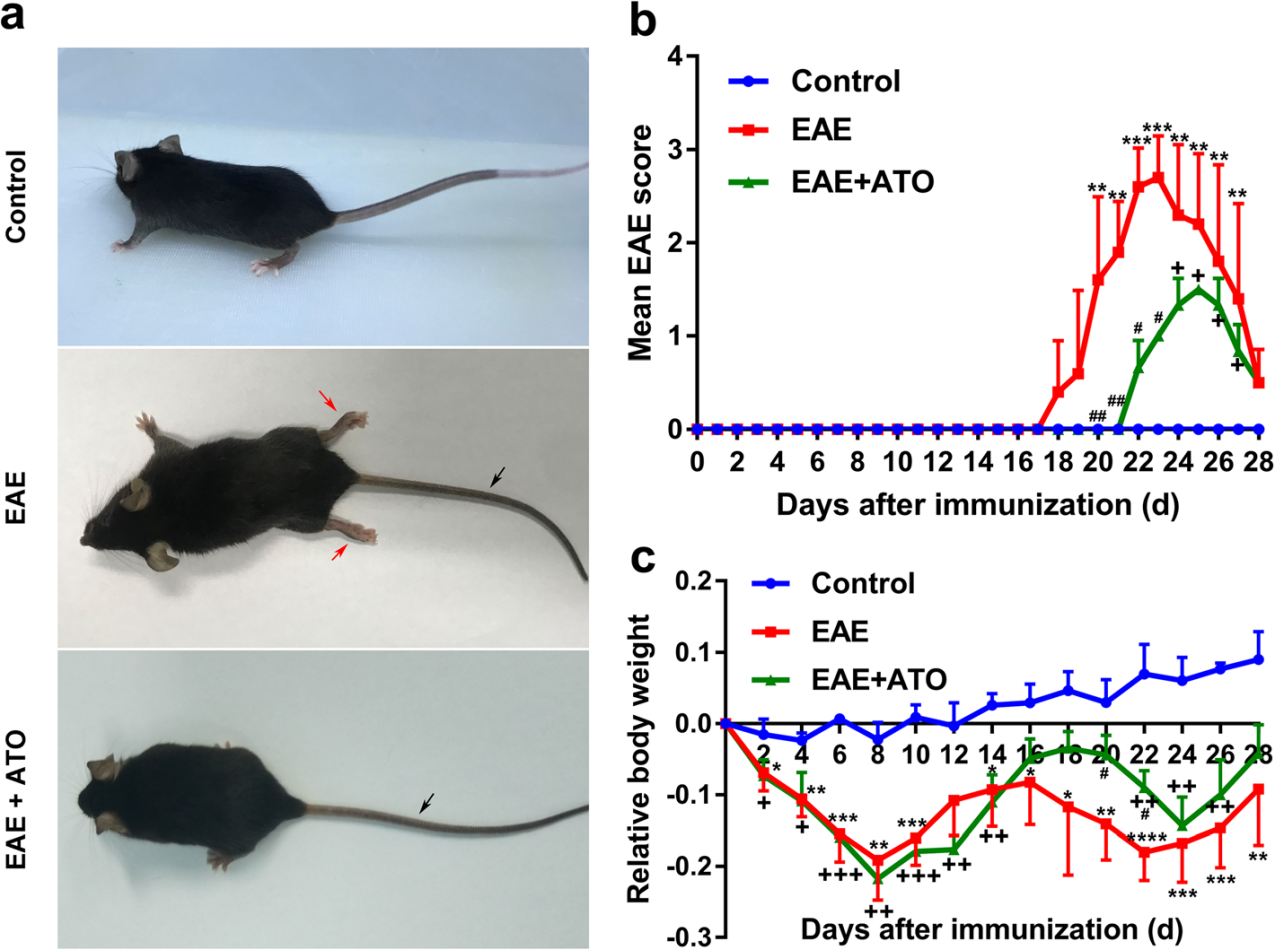
ATO ameliorates EAE progression in mice. **a** Representative images show behavioral symptoms of EAE mice in individual groups. Black arrow presents limp tail, and red arrow presents hind limb paralysis. The clinical scores (**b**) and changes in body weight (**c**) were recorded. *n* = 10 mice/group. Data are representative of three independent experiments. *p < 0.05, **p < 0.01, ***p < 0.001, and ****p < 0.0001 the EAE group vs. the control group; +*p* < 0.05, ++*p* < 0.01, and +++*p* < 0.001 the EAE+ATO group vs. the control group; #*p* < 0.05 and ##*p* < 0.01 the EAE+ATO group vs. the EAE group

We first explored whether ATO had a protective role on MOG_35–55_-induced EAE mice. Twenty-two days after immunization, EAE mice exhibited severe clinical signs with flaccid tail and complete paralysis of the hindlimbs; however, ATO-treated EAE mice only showed tail paralysis (Fig. 1a). EAE clinical score data showed that the onset of symptoms was later in ATO-treated mice (day 22) than that in EAE mice (day 18), and the maximal score (1.5) and mean score (1.02 ± 0.04) were lower in ATO-treated mice than those in the EAE mice (maximal [3.0] and mean score [1.86 ± 0.39]) (Fig. 1b). Additionally, weight loss was lower in ATO-treated mice than in EAE mice (Fig. 1c). These observations suggest that ATO may effectively reduce the severity of EAE in mice.

### ATO alleviated demyelination in the spinal cord of EAE mice

**Fig. 2:**
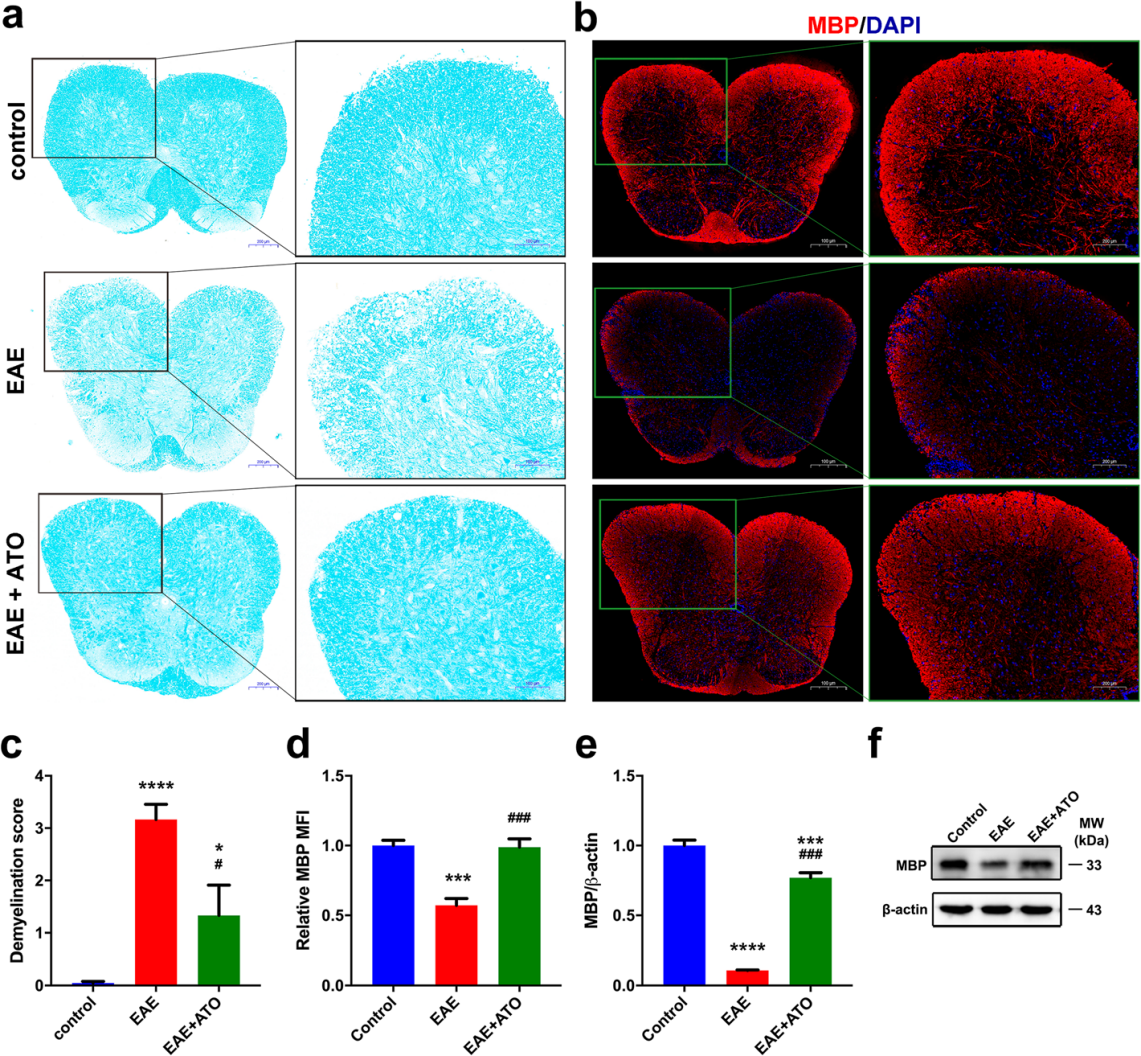
ATO alleviates demyelination in the spinal cord of EAE mice. Representative LFB staining sections (**a**) and demyelination score (**c**) of spinal cord (*n* = 9). Areas enclosed within black squares are magnified on the right. **b** Representative immunofluorescent staining sections of MBP in spinal cord. Areas enclosed within green squares are magnified on the right. **d** Mean fluorescence intensity analysis of MBP in **b** (*n* = 3). **e** The mRNA level of MBP in spinal cord was quantified by qRT-PCR (*n* = 3). **f** The protein level of MBP in spinal cord was analyzed by western blot. Data are representative of three independent experiments. *p < 0.05, ***p < 0.001, and ****p < 0.0001 vs. the control group; #*p* < 0.05 and ###*p* < 0.001 the EAE+ATO group vs. the EAE group

To further confirm the therapeutic effects of ATO on EAE mice, we measured demyelination in spinal cord using LFB staining. Results showed that the area of demyelination in the spinal cords of EAE mice was larger than that in ATO-treated EAE mice (Fig. 2a, c). Similarly, ATO-treated EAE mice had significantly increased expression of MBP, a structural protein of myelin, compared to that in the EAE mice (Fig. 2b, d–f). Overall, these findings suggest that ATO alleviated myelin damage associated with the progression of EAE.

### ATO reduced inflammation in the spinal cord of EAE mice

**Fig. 3:**
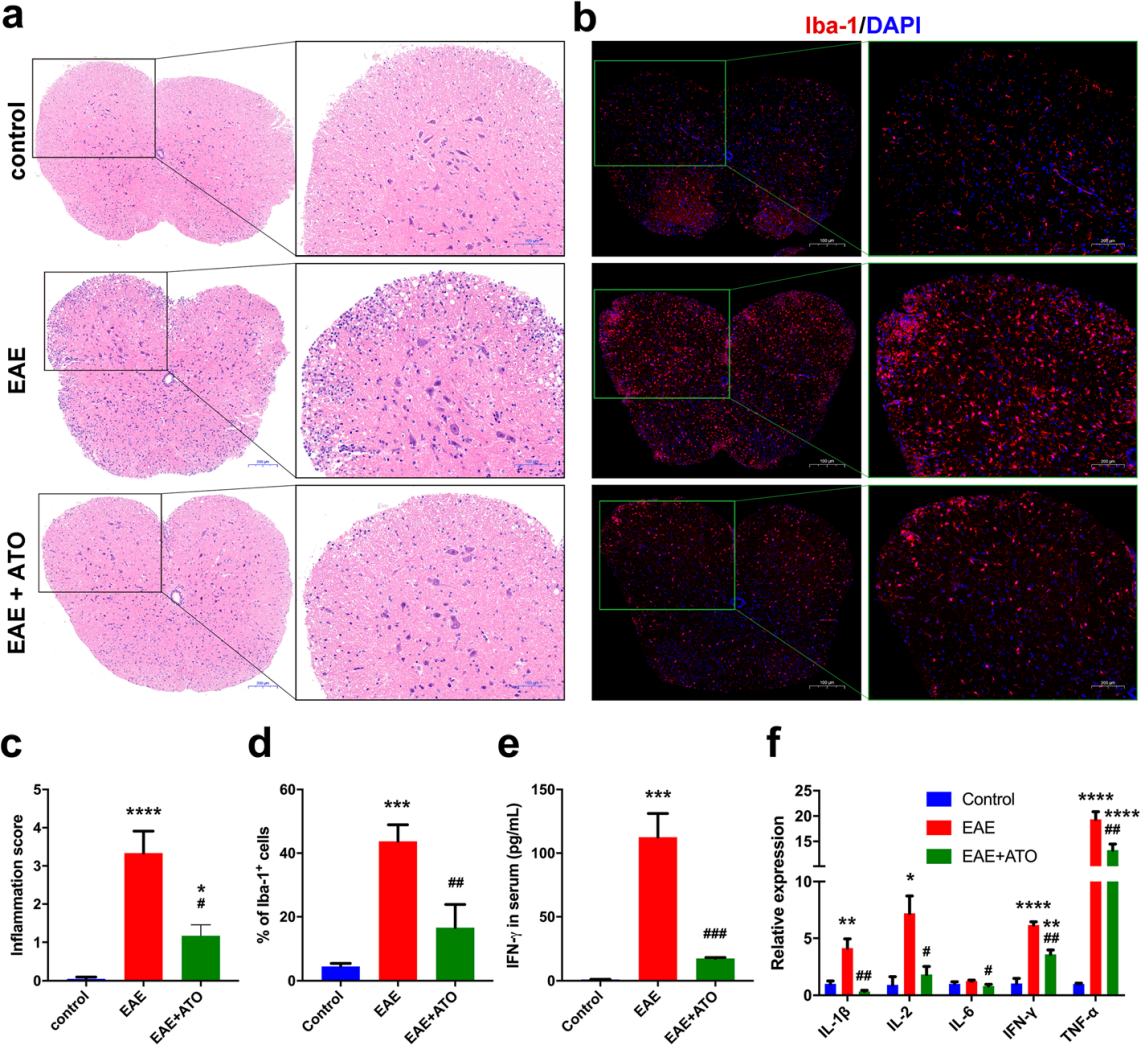
ATO reduces inflammation in the spinal cord of EAE mice. **a** Representative HE staining sections of spinal cord. Areas enclosed within black squares are magnified on the right. **c** Analysis of spinal cord infiltrates in **a** (*n* = 9). **b** Iba-1 immunofluorescent staining in spinal cord. Areas enclosed within green squares are magnified on the right. **d** Analysis of Iba-1^+^ cells in **b** (*n* = 3). **e** Concentration of IFN-γ in the serum was measured with the V-PLEX proinflammatory panel 1 kit (*n* = 3). **f** The mRNA levels of inflammatory cytokines in the spinal cord were determined through qRT-PCR (*n* = 3). Data are representative of three independent experiments. *p < 0.05, **p < 0.01, ***p < 0.001, and ****p < 0.0001 vs. the control group; #*p* < 0.05, ##*p* < 0.01, and ###*p* < 0.001 the EAE+ATO group vs. the EAE group

Since EAE is an autoimmune disease that is associated with severe neuroinflammation, we investigated whether ATO could decrease inflammatory cell infiltration, microglia activation, or inflammatory cytokine levels. Our results showed that EAE mice exhibited extensive inflammatory cell infiltration in the spinal cord, whereas ATO-treated EAE mice showed only mild infiltration (Fig. 3a, c). Additionally, microglia activation (as evidenced by Iba-1 expression) in the spinal cord was dramatically reduced in ATO-treated EAE mice compared to that in EAE mice (Fig. 3b, d). Moreover, treatment with ATO decreased the concentration of IFN-γ in the serum of EAE mice (Fig. 3e). The expression of inflammatory cytokines, such as IL-2, IFN-γ, IL-1β, IL-6, and TNF-α, was decreased in the spinal cord of ATO-treated EAE mice compared to that in EAE mice (Fig. 3f). Therefore, ATO appeared to reduce inflammatory cell infiltration and microglia activation during EAE progression.

### ATO reduced the number and proportion of CD4^+^ T cells in EAE mice by inducing apoptosis

**Fig. 4:**
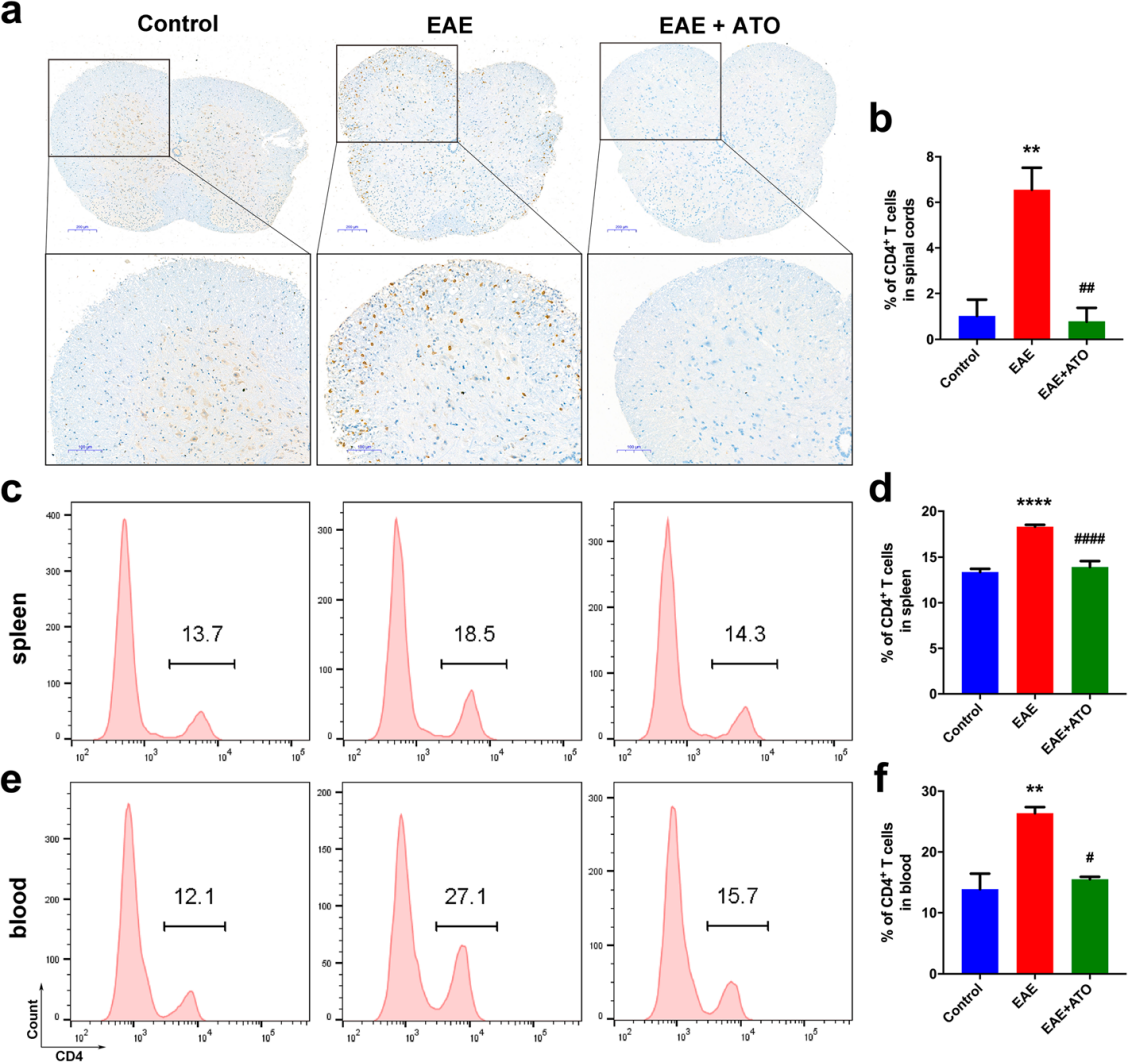
ATO reduces the number and proportion of CD4^+^ T cells in EAE mice. **a** Immunohistochemical staining of CD4 was performed in spinal cord. **b** Analysis of the infiltration of CD4^+^ T cells in **a** (*n* = 3). The percentage of CD4^+^ T cells in spleen (**c**) and peripheral blood (**e**) were determined by flow cytometry, respectively. **d**, **f** Analysis of CD4^+^ T cells in **c**, **e** (*n* = 3). Data are representative of three independent experiments. **p < 0.01 and ****p < 0.0001 vs. the control group; #*p* < 0.05, ##*p* < 0.01, and ####*p* < 0.0001 the EAE+ATO group vs. the EAE group.

Since CD4^+^ T cell-mediated neuroinflammation is considered to result in the initiation of EAE, we evaluated changes in the levels of CD4^+^ T cells in the spinal cord, spleen, and blood. EAE induction led to the prominent infiltration of CD4^+^ T cells in the spinal cord; however, this effect was counteracted following treatment with ATO (Fig. 4a, b). Similarly, treatment with ATO significantly reduced the proportions of CD4^+^ T cells in the spleen (Fig. 4c, d) and peripheral blood (Fig. 4e, f) compared to that in EAE mice.

**Fig. 5:**
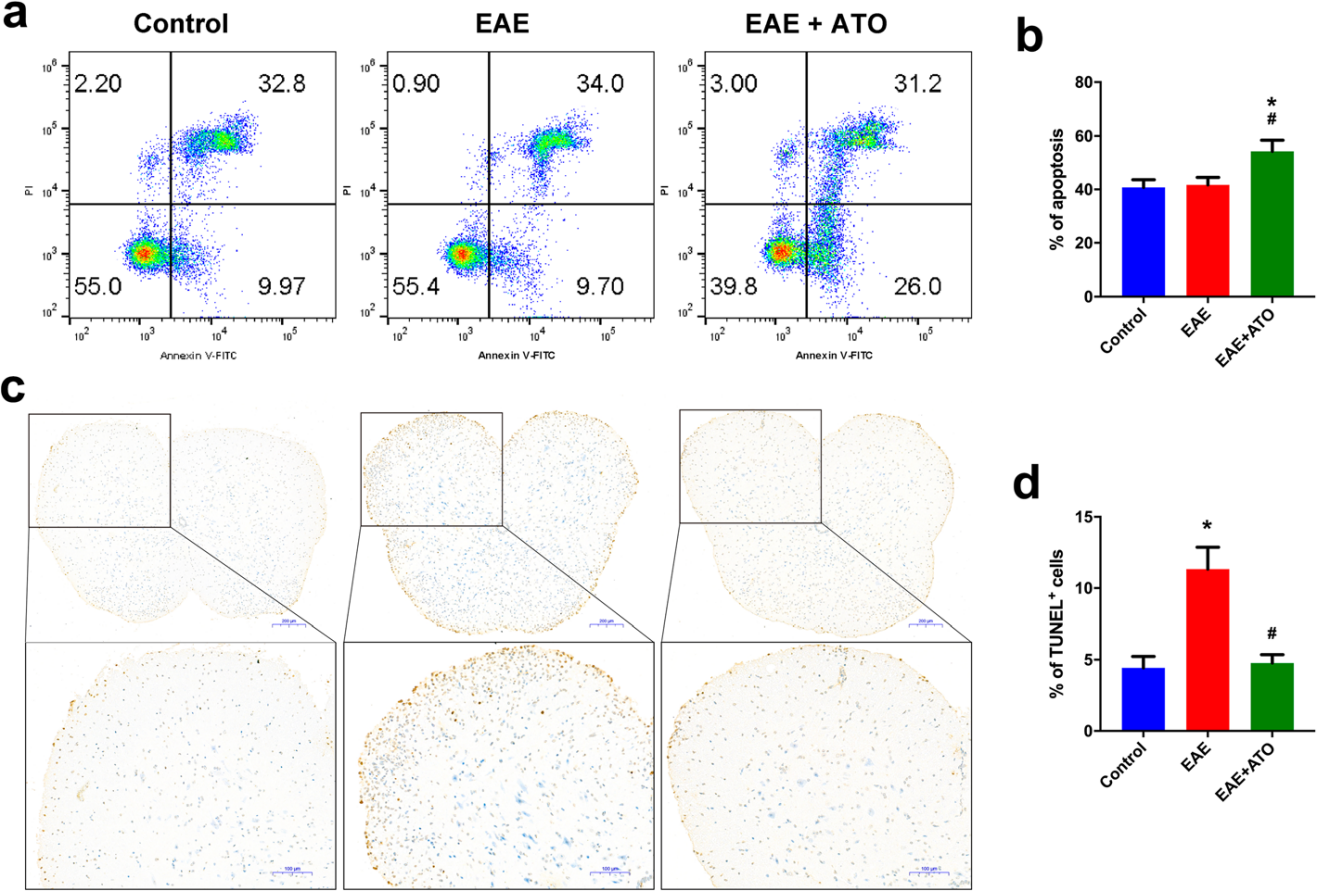
ATO induces CD4^+^ T cell apoptosis in EAE mice. **a**, **b** CD4^+^ T cells were isolated from the spleen using the mouse CD4+ T cell Isolation Kit. Annexin-V/PI staining was performed to analyze CD4^+^ T cell apoptosis (*n* = 3). **c** Representative TUNEL staining sections of spinal cord. Boxes denote magnified areas. **d** Analysis of TUNEL-positive cells in **c** (*n* = 3). Data are representative of three independent experiments. *p < 0.05 vs. the control group; #*p* < 0.05 the EAE+ATO group vs. the EAE group

Studies have reported that ATO induced apoptosis in a variety of cells, including T cells. Therefore, we investigated whether the ATO-reduced proportion and population of CD4^+^ T cells in EAE mice was related to apoptosis induction. The ratio of apoptotic CD4^+^ T cells in the spleen was significantly increased in ATO-treated EAE mice compared to that in the EAE mice (Fig. 5a, b). Additionally, the amount of TUNEL-positive signals was higher in EAE mice than that in ATO-treated EAE mice (Fig. 5c, d), showing that ATO decreased apoptosis in the spinal cord of EAE mice. Collectively, these results indicated that the decrease in CD4^+^ T cells in ATO-treated EAE mice may be attributed to apoptosis induction.

### ATO induced CD4^+^ T cell apoptosis through the mitochondrial pathway

**Fig. 6:**
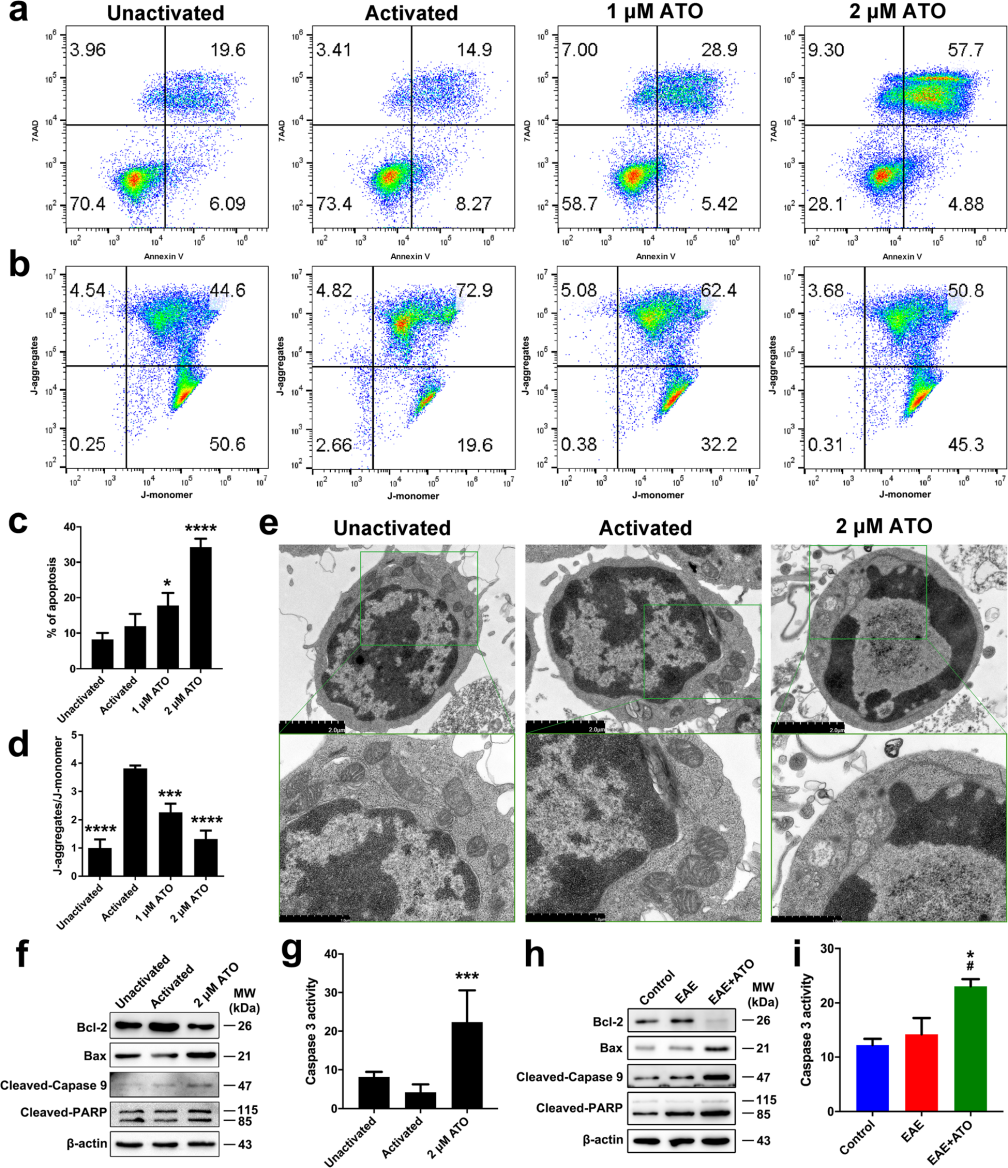
ATO induces CD4^+^ T cell apoptosis through the mitochondrial pathway. CD4^+^ T cells were sorted from the spleen of normal mice and cultured with Con A or ATO (0, 1, and 2 μM) for 24 h. **a**, **c** Annexin V/PI was performed to assess CD4^+^ T cell apoptosis (*n* = 3). **b**, **d** The mitochondrial membrane potential was monitored by loading with JC-1 probe. J-aggregates indicates high membrane potential, and J-monomers indicates low membrane potential (*n* = 3). **e** Mitochondrial ultrastructure as observed by TEM. Boxes denote magnified areas. **f** The protein level of Bax, Bcl-2, cleaved-Caspase 9, and cleaved-PARP was analyzed by western blot. **h** On day 22 post-immunization, CD4^+^ T cells isolated from the spleen were used to extract total protein. The protein levels of Bax, Bcl-2, cleaved-Caspase 9, and cleaved-PARP in CD4^+^ T cells were detected by western blot. **g**, **i** The activity of Caspase 3 was determined with the Caspase 3 Activity Assay kit (*n* = 3). Data are representative of three independent experiments. *p < 0.05, ***p < 0.001, and ****p < 0.0001 vs. the activated or control group; #*p* < 0.05 the EAE+ATO group vs. the EAE group

To investigate the underlying mechanism of ATO-induced apoptosis in vitro, CD4^+^ T cells were isolated from naïve C57BL/6 mice and cultured for 24 h. As shown in Fig. 6a, c, ATO significantly increased the ratio of apoptotic cells in a dose-dependent manner. The mitochondrial membrane potential was high in activated CD4^+^ T cells, whereas ATO treatment diminished the J-aggregates and increased the J-monomers (Fig. 6b, d). Moreover, transmission electron microscopic examination showed that mitochondria swelling, decrease or disappearance of mitochondria crista, and vacuolization were observed in ATO-treated groups (Fig. 6e). Meanwhile, the level of pro-apoptotic proteins, such as cleaved-Caspase 3, Bax, cleaved-Caspase 9, and cleaved-PARP, was significantly increased in ATO-treated EAE mice; however, ATO decreased Bcl-2 expression (Fig. 6f, g). Moreover, consistent with the results observed in vitro, ATO treatment significantly increased the level of pro-apoptotic proteins in the spleen of EAE mice, while decreased Bcl-2 expression (Fig. 6h, i). These data indicated that ATO induced CD4^+^ T cell apoptosis through the mitochondrial pathway both in vitro and in vivo.

### No toxicity signs were evident in ATO-treated mice

**Fig. 7:**
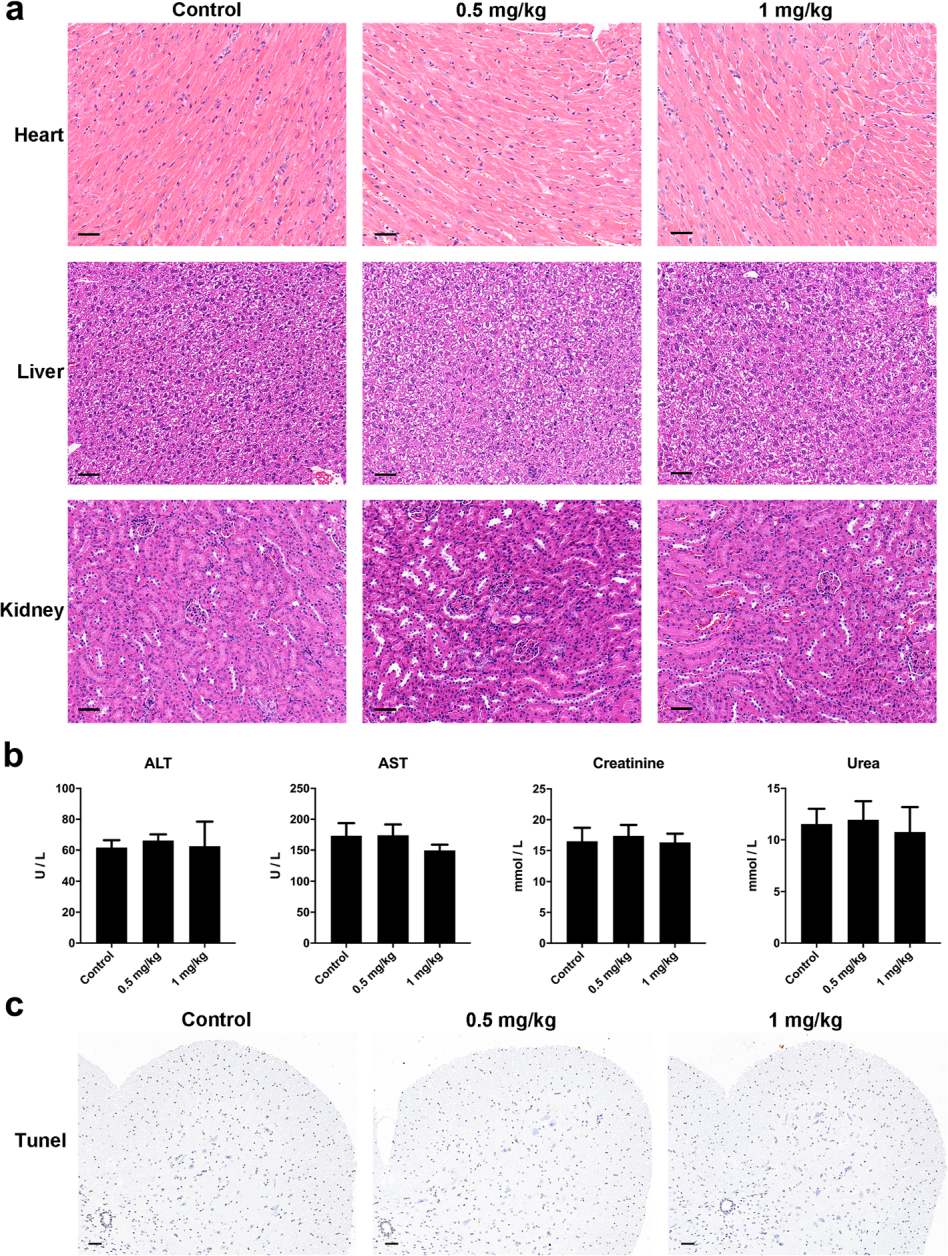
No signs of toxicity were observed in ATO-treated mice. Normal mice were administered intraperitoneal injections of normal saline, ATO (0.5 mg/kg/day) or ATO (1 mg/kg/day) for 20 days. **a** Heart, liver, and kidney samples were collected for HE staining. Scale bar = 50 μm. **b** The levels of ALT, AST, creatinine, and urea in the serum were measured using an automated biochemical analyzer. **c** Representative TUNEL staining sections of the spinal cord. Scale bar = 100 μm. *n* = 5 mice/group. Data are representative of three independent experiments. ALT, alanine aminotransferase; AST, aspartic aminotransferase

To determine whether the administration of ATO had any toxic effects on mice, we analyzed the functions of liver and kidney after consecutive intraperitoneal injections of ATO for 20 days. The results of HE staining showed that there were no obvious abnormalities in the heart, liver, or kidney of mice following ATO treatment compared with control mice (Fig. 7a). Consistently, no significant differences in the levels of ALT, AST, creatinine, and urea were observed between ATO-treated mice and control mice, even at a dose of 1 mg/kg/day (Fig. 7b). Moreover, treatment with ATO did not result in evident TUNEL-positive signals in the spinal cord (Fig. 7c). These results suggested that ATO had no adverse effect on liver or kidney function at a dose of 0.5 or 1 mg/kg/day and did not induce apoptosis in the spinal cord.

## Discussion

Despite recent advances with regard to DMTs for the treatment of MS patients, the overall therapeutic effect with MS has not yet been maximized, and developing new and safe drugs for the treatment of MS remains an important goal. EAE is a classic mouse model used for the study of MS. Studies reported that ATO demonstrates anti-inflammatory or immunosuppressive effects in a variety of disease settings, including leukemia [16], asthma [18], human lupus-like syndrome [19], and graft-versus-host disease [20]. However, it remains unclear whether ATO has a therapeutic effect on EAE. To the best of our knowledge, this is the first to attempt to explore the possibility of using ATO to ameliorate EAE in mice. Gogoleva et al. had reported that EAE in C57BL/6 mice is characterized by more severe tissue damage of the spinal cord than of the brain [24]. Therefore, in the present study, the spinal cord was isolated and used for experimental analysis. Our results suggested that ATO delayed the onset of EAE, alleviated the clinical signs and severity of EAE in mice, reduced neuroinflammation, and attenuated demyelination.

Autoreactive CD4^+^ T cells play a major role in the initiation and orchestration of EAE [25]. The activated CD4^+^ T cells migrate from the periphery to the CNS, where the cascade of inflammatory reactions is initiated by secreting cytokines and chemokines. Therefore, the apoptosis induction of CD4^+^ T cells may contribute to ameliorating EAE. Studies reported that drugs such as glatiramer acetate [26] and interferon-β [27] treat MS by inducing the peripheral T cells apoptosis. Similarly, results from our study demonstrated that ATO induced CD4^+^ T cell apoptosis in the spleen. Cell apoptosis could be triggered by the mitochondrial pathway, the endoplasmic reticulum stress pathway, or the death receptor-mediated extrinsic pathway. Among these pathways, the mitochondrial pathway can be activated by caspase cascades and Bcl-2 family members in the mitochondria [28]. Other studies have reported that ATO-induced apoptosis is attributed to the downregulation of Bcl-2, the upregulation of Bax, and the reduction of the mitochondrial membrane potential [29]. Results from our study support these findings and demonstrate that ATO induced alterations in the protein level of Bcl-2 and Bax and decreased the mitochondrial membrane potential in CD4^+^ T cells. Additionally, our results suggest that ATO decreased the proportion of CD4^+^ T cells in the spleen and peripheral blood, probably due to apoptosis induction; thus, the population of CD4^+^ T cells that infiltrated the CNS was also reduced. Indeed, we found that treatment with ATO decreased the number of CD4^+^ T cells infiltrating the white matter. Therefore, these data indicate that ATO treatment could induce CD4^+^ T cells apoptosis through the mitochondrial pathway, thus inhibiting the infiltration of CD4^+^ T cells into the CNS, thereby delaying the onset of EAE and effectively reducing the severity of EAE in mice.

Some pro-inflammatory cytokines secreted by activated CD4^+^ T cells in the CNS could attract various immune cells into the CNS, gradually aggravating CNS demyelination. Thus, inhibition of the release of some proinflammatory cytokines is an effective strategy to attenuate EAE clinical symptoms. IFN-γ and IL-6 can induce the expression of chemokines and adhesion molecules, recruiting leukocytes migrating into the CNS [30, 31]. In the CNS, IL-1β can recruit and activate lymphocytes by acting on astrocytes and CNS endothelial cells [32]. TNF-α produced by infiltrated macrophages can exacerbate the severity of EAE by promoting inflammatory infiltrates and disrupting the blood-brain barrier [33]. Our data demonstrated that treatment with ATO decreased the mRNA levels of inflammatory cytokines in the spinal cord and reduced the concentration of IFN-γ in the serum of EAE mice. Consistent with our findings, studies have reported that ATO reduced inflammation in splenocytes of MRL/*lpr* mice and human lupus peripheral blood mononuclear cells by reducing the expression levels of INF-γ [34]. Collectively, the therapeutic effects of ATO in our study may be partially due to the lower expression levels of proinflammatory cytokines, such as IL-2, IFN-γ, IL-1β, IL-6, and TNF-α.

Excessive activation of microglia induces neuroaxonal injury [35]. As the resident macrophages of the CNS, microglia activation is secondary to infiltrating CD4^+^ T cells and a hallmark of demyelinated lesions [36, 37]. Even though the role of microglia in autoimmune diseases such as EAE remains undefined, studies have reported that microglia activation results in damage to myelin, axonal and neuron, probably mediated by high levels of oxidative stress and iNOS as well as the excessive release of some cytotoxic mediators [38, 39]. In this study, a treatment with ATO reduced the expression level of Iba-1, indicating that ATO decreased microglial activation, which also suggested that ATO alleviated demyelination in the spinal cord of EAE mice. Consequently, part of the therapeutic effects of ATO in EAE mice may be due to its ability to reduce the proportion of peripheral CD4^+^ T cells and microglia activation, ultimately inhibiting demyelination.

The clinical application of ATO may be limited by its adverse effects on healthy tissues, including cardiotoxicity, hepatotoxicity, and nephrotoxicity. However, a 10-year follow-up study suggested that none of the side effects were severe enough to discontinue treatment. Their observations suggested that the long-term use of ATO in APL patients was safe and not associated with any major side effects [40]. Moreover, studies from Lo-Coco et al. and Burnett et al. have shown that the hepatotoxicity is usually reversible and may be successfully managed with the temporary cessation of or a decrease in ATO; there have been no reports of fatal hepatic failure in clinical trials [41, 42]. Furthermore, increasing evidence has indicated that multiple drugs could be used for the inhibition of cardiotoxicity induced by ATO, such as salvianolic acid A, omega-3 fatty acid, sorbus pohuashanensis, resveratrol, genistein, and metallothionein [43]. ATO-mediated cardiotoxicity could be reduced by combining it with other drugs. Therefore, the toxic side effects of ATO as a clinical drug are relatively controllable. Zheng et al. [44] and our previous work [45, 46] have suggested that in the allogeneic heart transplantation mouse model, when the dose of ATO is less than 5 mg/kg/day, no abnormalities are observed in the liver, kidney, and lung. Zhang et al. have reported that treatment with 1 mg/kg/day ATO for 14 days did not cause damage to the hearts of the mice [47]. These findings suggest that ATO has no toxic side effects on the heart, liver, kidney, and lungs at doses under 5 mg/kg/day in animal models.

There appears to be a contradiction between the therapeutic effect of ATO on nervous system diseases such as EAE and the reported ATO-induced neurotoxicity, which warrants further investigation. However, the therapeutic effect and toxic side effects of ATO are closely associated with the administrated dose [48]. Récher et al. have shown that mice intraperitoneally injected with ATO (5 mg/kg/day) for two consecutive weeks (5 days per week) had a blood ATO concentration of 0.23 μM after 2 weeks of treatment [49]. Lu et al. found that 1 μM ATO did not induce neuronal cell apoptosis in vitro [50]. Thus, we believe that a lower ATO dose than that used in Récher et al.’s study, that is, 0.5 mg/kg/day, will not result in neurotoxicity in mice. Indeed, we did not observe any TUNEL-positive signaling in the spinal cord after mice were administrated 0.5 or 1 mg/kg/day ATO for 20 days. Therefore, in a certain concentration range, ATO has no adverse effects on the CNS.

In this study, mice in the EAE + ATO group were intraperitoneally injected with ATO at a dose of 0.5 mg/kg/day for 8 days based on the following considerations. In a murine model of asthma, treatment with 2.5 mg/kg/day of ATO for 7 days alleviates airway hyperresponsivity and eosinophilia [18]. In MRL/*lpr* mice, treatment with 5 mg/kg/day of ATO for 2 months inhibited autoreactive lymphocytes and blocked the progression of autoimmune diseases [19]. In a mouse allogeneic islet transplantation model, treatment with 1 mg/kg/day of ATO twice per day significantly prolonged the survival of the islet allograft [22]. A recent study suggested that ATO (1 mg/kg/day) suppressed acute graft-versus-host disease in mice [20]. Based on the above, 2 doses of ATO (0.5 and 1 mg/kg) were studied to determine the optimal dose in pre-experiments. Treatment with ATO (0.5 or 1 mg/kg) for 8 days had similar effects in ameliorating EAE. Since 0.5 mg/kg was the lowest therapeutic dose of ATO against EAE, it was used for subsequent experiments. Furthermore, treatment with 0.5 or 1 mg/kg ATO did not result in any adverse effects on the heart, liver, and kidney of mice. Therefore, the dose of ATO used in this study is safe and effective.

The successful application of ATO in the mouse EAE model may contribute to treating other immune diseases such as MS. Several comorbidities often occur in MS patients, such as cancer and autoimmune diseases, which limit the choice of DMTs and reduce quality of life [4]. Thus, the development of drugs that possess both anti-tumor and anti-autoimmunity properties may also benefit MS patients. At this point, ATO may contribute to the management of these comorbidities due to its antineoplastic [51–54] and anti-inflammatory [20, 22, 55] properties, thereby improving the efficiency of DMTs therapy. Additionally, as a traditional Chinese medicine, ATO has been used for thousands of years [56] and is the first-line drug for the treatment of APL in clinical settings [16]. The new use of old drugs is an effective way of developing new drugs. Although evidence showing that arsenic may be associated with the risk of developing MS or MS progression [57–60], the types of arsenic compounds involved in MS pathogenesis remain unclear. Our results suggested that ATO could effectively ameliorate symptoms of EAE in MOG-induced EAE mice. Moreover, only one case was reported in which inorganic arsenic (Fowler’s solution) had been used in the past century to treat MS [61]. Therefore, further experiments incorporating a larger population are required to better understand the effect of ATO in the treatment of MS in the future.

Although our results suggest that ATO has a good therapeutic effect on EAE in mice, this study has some limitations. EAE mice in our study developed a relatively mild monophasic disease course instead of the more severe chronic progressive form that is usually observed for such robust induction conditions. Since both genetic and environmental factors likely contribute to the development of EAE [24], this may be due to the housing of mice in SPF conditions. Despite the pathogenesis of MS not being fully elucidated, CD4^+^ T cells, CD8^+^ T cells, B cells, and other immune cells are likely to be involved [1]. Since EAE is initiated and orchestrated by autoreactive CD4^+^ T cells, we focused on the CD4^+^ T cells in both in vivo and in vitro experiments. Therefore, it is necessary to use other suitable animal models to explore the role of CD8^+^ T cells, B cells, and other immune cells in the context of the curative effect of ATO on EAE in future work. Moreover, the effects of ATO on regulatory, anti-inflammatory cell types or cytokines in EAE mice may be a focus of our future studies.

## Conclusions

To the best of our knowledge, our study is the first to suggest that ATO ameliorated EAE in C57BL/6 mice by inducing CD4^+^ T cell apoptosis via the mitochondrial pathway. Moreover, the administration of ATO did not cause adverse health effects in mice. Our findings may facilitate the clinical application of ATO for the treatment of MS or other autoimmune diseases.

## Supplementary information

**Additional file 1: Figure S1.** Representative examples of LFB stained histological sections illustrating the different demyelination scores.

## Data Availability

All data used in this study are available from the corresponding author on reasonable request.

## Abbreviations

MS: Multiple sclerosis
ATO: Arsenic trioxide
EAE: Experimental autoimmune encephalomyelitis
LFB: Luxol fast blue
TEM: Transmission electron microscope
qRT-PCR: Quantitative real-time PCR
CNS: Central nervous system
Th: T helper
FITC: Fluorescein isothiocyanate
PBS: Phosphate-buffered saline
MBP: Myelin basic protein
TUNEL: TdT-mediated dUTP nick end labeling
IL-1β: Interleukin-1β
MOG: Myelin oligodendrocyte glycoprotein
IL-2: Interleukin-2
CFA: Complete Freund’s adjuvant
IL-6: Interleukin-6
HE: Hematoxylin and eosin
IFN-γ: Interferon gamma
TNF-α: Tumor necrosis factor-α
PI: Propidium iodide
ANOVA: Analysis of variance
SD: Standard deviation
ALT: Alanine aminotransferase
AST: Aspartic aminotransferase
DMTs: Disease modifying therapies
