# Supplementary figures and images for: Arsenic trioxide ameliorates experimental autoimmune encephalomyelitis in C57BL/6 mice by inducing CD4+ T cell apoptosis
Source: J Neuroinflammation. 2020 May 6;17:147. doi: 10.1186/s12974-020-01829-x (PMC7201567; doi:10.1186/s12974-020-01829-x)

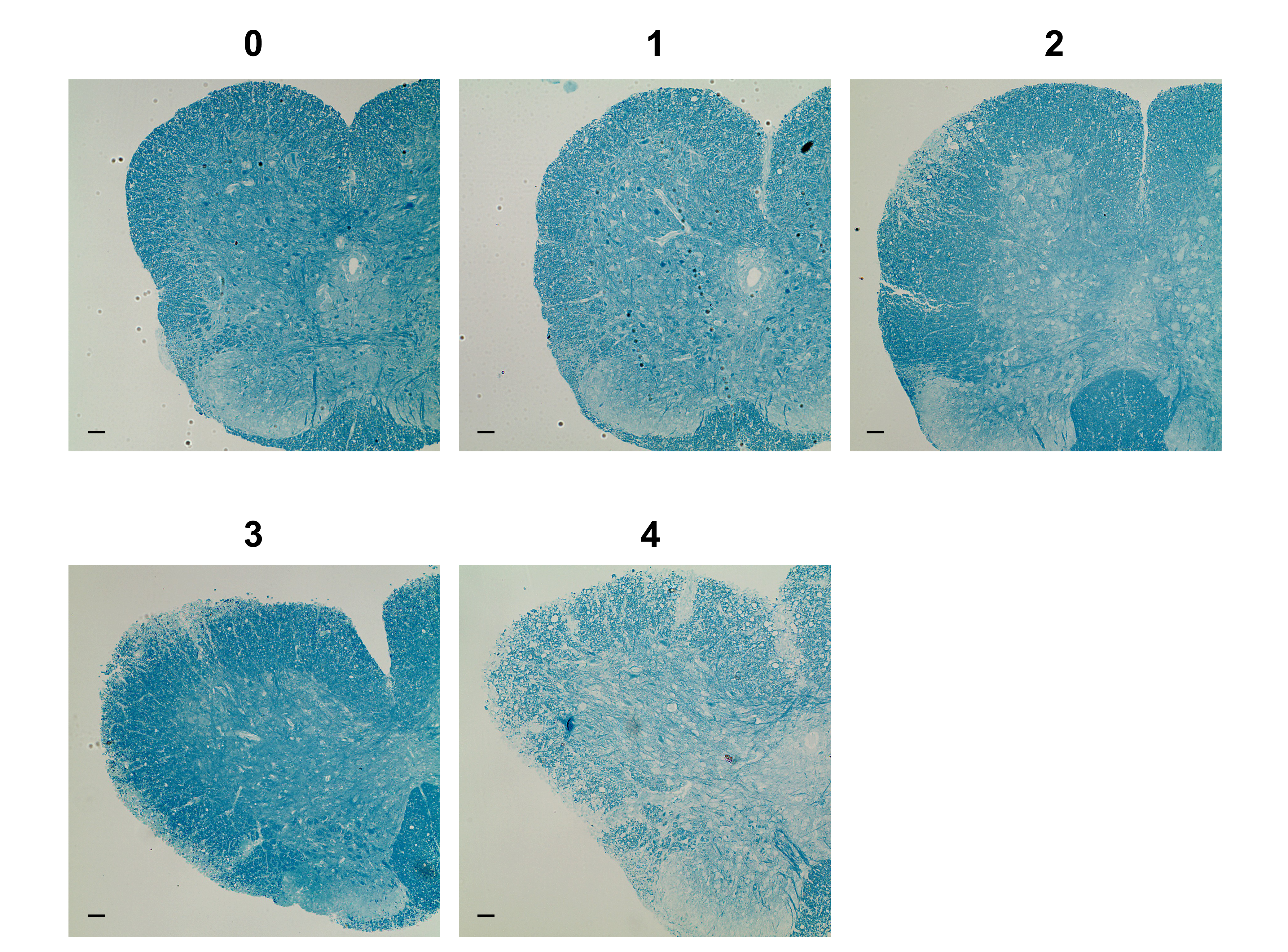

Supplement: Supplementary file 1 — Additional file 1: Figure S1. Representative examples of LFB stained histological sections illustrating the different demyelination scores. [file 12974_2020_1829_MOESM1_ESM.tif]
